# Mechanical and chemical surface treatment enhances bond strength between zirconia and orthodontic brackets: an in vitro study

**DOI:** 10.1038/s41405-023-00180-6

**Published:** 2023-12-08

**Authors:** Nareudee Limpuangthip, Atikom Surintanasarn, Ploylada Vitavaspan

**Affiliations:** 1 Department of Prosthodontics, Faculty of Dentistry, Chulalongkorn University, Bangkok, Thailand; 2 Private dental clinic, Bangkok, Thailand

**Keywords:** Dental materials, Fixed prosthodontics

## Abstract

**Objectives:**

To assess the shear bond strength (SBS) between metal orthodontic brackets and zirconia after receiving different mechanical and chemical surface treatments, and different types of resin adhesive. The failure mode of each treatment protocol was also evaluated.

**Materials and methods:**

The present in vitro experimental study consisted of six surface treatment protocols with two different resin adhesives. One-hundred and forty-four rectangular-shaped 3 mol% yttrium-stabilized tetragonal zirconia polycrystal blocks were milled, sintered, and embedded in acrylic resin. They were randomly divided into three mechanical (none, air abrasion, and bur grinding) and two chemical surface treatment conditions (no primer and Z-primer). The specimens were divided into two groups according to the resin adhesive received: self-cured (RelyX U200) and light-cured adhesives (Transbond XT). The SBS between the metal bracket and zirconia was tested using a universal testing machine (1-mm/min crosshead speed), and the failure mode was evaluated. Differences in SBS and failure mode were analyzed using Welch ANOVA followed by post-hoc comparison and Fisher’s Exact test, respectively.

**Results:**

Bur grinding produced the highest SBS, followed by air abrasion. Z-primer application typically provided a higher SBS regardless of resin adhesive used (*p* < 0.001). Without primer application, RelyX U200 provided a higher SBS than Transbond XT (*p* < 0.001). After grinding, using Z-primer and RelyX U200 resulted in a higher SBS than no primer and using Transbond XT (*p* < 0.001). Adhesive failure at the zirconia–adhesive interface occurred only when Transbond XT was applied without bur grinding, and when using Transbond XT after grinding, but no Z-primer application.

**Conclusion:**

Bur grinding combined with applying an MDP-containing primer and resin adhesive enhances the SBS between zirconia and metal orthodontic brackets.

## Introduction

All-ceramic materials become more popular in fixed dental prosthodontics because they provide a high esthetic appearance with optimal strength [1, 2]. Monolithic zirconia is the material of choice for all-ceramic restorations because it provides higher mechanical strength than lithium disilicate glass ceramic, and demonstrates less porcelain chipping and fracture compared with layered zirconia [2, 3]. However, the disadvantage of monolithic zirconia is its poor bond strength to other materials including composite resin and metal [4].

Because the demand for orthodontic treatment in adults has increased, the orthodontic brackets frequently need to be bonded to enamel and different types of ceramic restorations, including zirconia [5, 6]. However, there is a higher occurrence of debonding between orthodontic brackets and zirconia restorations compared with bonding to enamel [7]. Bracket bonding is a critical step in orthodontic treatment because orthodontic bracket debonding can delay orthodontic treatment completion, and increase the risk of a detached bracket going into the patient’s throat [8].

The factors affecting the adhesion of an orthodontic bracket to zirconia includes the bracket materials [9, 10] and surface treatment conditions [4, 10–17], comprising mechanical and chemical protocols. Mechanical surface treatments include polishing with a prophy cup and pumice [11], air abrasion with alumina oxide particles [11, 14, 15], surface roughening [16], and laser irradiation [10, 13]. Moreover, chemical surface treatments include etching with various acids, such as orthophosphoric [14] and hydrofluoric acid [11, 13], and primer application [11, 12, 17]. In addition, the significant impact of combined mechanical and chemical method such as tribochemical silica coating [10, 13], and types of resin adhesive [16] on adhesion has been reported. Although a recent systematic review demonstrated the positive effect of air abrasion and laser treatment of zirconia for bonding with orthodontic brackets [4], due to the heterogeneity among studies, it has not been determined which surface treatment methods should be recommended as the standard protocol. Furthermore, few studies have reported the combined effects of mechanical and chemical surface treatment, as well as resin adhesive type on the adhesion between orthodontic brackets and zirconia restorations.

Therefore, the objective of this study was to assess the shear bond strength (SBS) between metal orthodontic brackets and zirconia after receiving different mechanical and chemical surface treatments, as well as different types of resin adhesive. The failure mode of each condition was also evaluated. The null hypothesis was that neither mechanical and chemical surface treatment nor resin adhesive type would have an impact on the SBS between metal orthodontic brackets and zirconia.

## Materials and methods

**Fig. 1:**
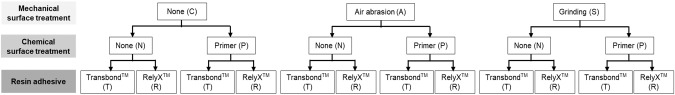
Flow diagram of the surface treatment. Flow diagram of the surface treatment. Specimens were randomly divided into three mechanical (no surface treatment, air abrasion, and bur grinding), two chemical surface treatment conditions (no primer and zirconia primer application), and two types of resin adhesives (light-cured [Transbond^TM^] and self-cured [RelyX^TM^]).

The present in vitro experimental study consisted of six surface treatment protocols with two different resin adhesives. Table 1 presents the materials used in this study, and the flow diagram is demonstrated in Fig. 1. The sample size was estimated using G*Power program version 3.1.9.4 (Heinrich-Heine-Universität Düsseldorf) based on the results of a previous study that showed an effect size of 1.56 for the SBS between zirconia and metal brackets after using a zirconia primer and adhesive [18]. Based on the difference between two groups using the independent *t*-test and accounting for a 20% failure rate during specimen preparation, a minimum of 12 samples per group were required to achieve 90% power and a 5% type I error.

**Table 1:** Materials used in this study.

Material type	Brand	Manufacturer	Main composition
Mechanical surface treatment			
Aluminum oxide particle (Al_2_O_3_)			50-µm aluminum oxide particle
Diamond bur	Intensive	Intensive SA, Grancia, Switzerland	106-µm diameter diamond grit size
Chemical surface treatment			
Primer	Z-Prime^TM^ Plus	Bisco, Inc. USA	- Bis-GMA - MDP - 2-hydroxyethyl methacrylate, ethanol
Resin adhesives			
Dual cure self-adhesive	RelyX^TM^ U200	3M EPSE, USA	Base: - methacrylate monomers, and those containing phosphoric acid groups - silanated fillers, initiators, stabilizers, rheological additives. Catalyst: - methacrylate monomers - silanated fillers, alkaline stabilizers, rheological additives.
Light cure self-adhesive	Transbond^TM^ XT	3M EPSE, USA	Adhesive: - bis-GMA, silane, - bisphenol A bis (2-hydroxyethyl ether) dimethacrylate - diphenyliodonium hexafluorophosphate Primer: - bis-GMA, TEGDMA - triphenyl antimony, 4-(dimethylamino)-benzeneethanol - DL-camphorquinone, hydroquinone

*Bis-GMA* bisphenol-A-diglycidylmethacrylate, *MDP* 10-methacryloxydecyl dihydrogen phosphate, *TEGDMA* triethyleneglycol dimethacrylate.

### Specimen preparation

A total of 144 rectangular pre-sintered 3 mol% yttrium-stabilized tetragonal zirconia polycrystal (YTZP) blocks (KATANA Zirconia; Kuraray Noritake Dental Inc) were milled into 8 × 8 × 2 mm^3^ blocks using a low-speed saw (Isomet 1000; Buehler, USA). The blocks were polished with #800 and #1200 grit sandpaper attached to a polishing machine (Minitech 233; Presi, Grenoble) at a polishing speed of 250 rpm with a 50-N force for 1 min. After sintering in a high-temperature furnace (LHT 04/16; Nabertherm GmbH) at 1,500 ^o^C for 2 h, the sintered YTZP blocks were embedded in dental gypsum (Vel-MixTM Die Stone Gypsum; Kerr) in a polyvinyl chloride pipe. The specimen’s surface was re-polished with #800 and #1200 sandpaper attached to a polishing machine at a polishing speed of 250 rpm for 1 min. The surface roughness of the specimens was measured using a contact profilometer (Talyscan 150) to ensure that the baseline surface roughness of the specimens was similar.

### Surface treatment and bracket bonding

**Fig. 2:**
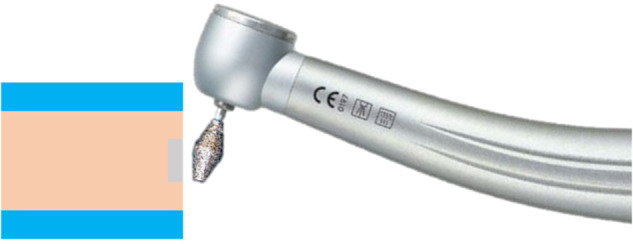
Bur grinding of the zirconia specimen. A double cone-shaped diamond bur aligned with a flat surface parallel to the specimen surface was attached to a high-speed airotor, which was connected to the computer numerical control.

The prepared specimens were randomly divided into three mechanical (no surface treatment, air abrasion, and grinding) and two chemical surface treatment conditions (no primer and primer application). In the air abrasion protocol, the YTZP surface was sandblasted with 50-µm aluminum oxide particles at a 10-mm distance from the surface using a sandblasting machine (Vario Basic®; Renfert, Germany) at 0.25-MPa pressure for 15 sec, perpendicular to the specimen’s surface. To grind the YTZP surface, a standard double cone-shaped diamond bur (Intensive SA, Grancia, Switzerland) was aligned with a flat surface parallel to the specimen surface (Fig. 2). The bur was attached to a high-speed airotor (Alegra dental turbine; W&H company), which was connected to the computer numerical control (CNC) used to control the force and direction of the bur. To grind the specimen’s surface, the CNC moved from left-to-right while the airotor remained fixed, and the bur was changed after 12 specimens. After the mechanical surface treatment, the specimens were cleaned with distilled water in an ultrasonic cleaner for 5 min, and dried with absorbent paper. A 50-µm clear adhesive tape with a 2.38-mm diameter window was attached onto each specimen to control the adhesive thickness and area [19, 20]. To perform the chemical surface treatment, a zirconia primer (Z-Prime^TM^ Plus) was applied onto the specimen’s surface using a microbrush, which was changed for each specimen, and dried with an air syringe for 5 sec [21].

After the surface treatment, the specimens in each group were randomly divided into 2 groups (*n* = 12 per group) according to the adhesive type received; light-cured (Transbond^TM^ XT) and self-cured (Rely X^TM^ U200; 3 M ESPE) resin adhesives. The resin cement was mixed according to the manufacturer’s instructions and applied on the specimen’s surface. The metal bracket was adhered to the zirconia using a 1-kg static load with the metal bracket surface perpendicular to the applied force. After removing the excess cement with a microbrush, the bonded specimens were light-cured with a light-curing unit (Elipar™ S10 LED Curing Light; 3 M ESPE) on four sides of the specimens for 20 sec on each side. The specimens were stored in distilled water at 37^o^C in an incubator for 24 h prior to the SBS test.

### Shear bond strength test

The SBS was determined using a universal testing machine (EZ-S 500 N, Shimadzu Corporation, Kyoto, Japan) according to a protocol modified from ISO 29022:2013 [22]. The head was aligned parallel to the junction between the zirconia and metal bracket, and applied with a crosshead speed of 1 mm/min until debonding occurred. The SBS was recorded in megapascals (MPa) from the applied force in newtons (N) divided by the bonding surface area (mm^2^).

### Mode of failure

The bonded surfaces of the zirconia and bracket sides were examined using a stereomicroscope at 45x magnification to determine the mode of failure, which was categorized into 4 types: [23] adhesive failure at the zirconia-resin interface (at least 70% of the zirconia surface detached), adhesive failure at the metal-resin interface (at least 70% of the metal surface detached), cohesive failure (at least 70% within the resin adhesive), or mixed failure (failure at the zirconia-resin adhesive interface and 30–70% within the resin adhesive). The zirconia surface was also assessed using an Adhesive Remnant Index (ARI) score, where each specimen was scored as follows: 0 for no remaining composite, 1 for less than 50% remaining, 2 for more than 50% remaining, and 3 for all remaining composite with a distinct impression of the bracket base [11]. The images were captured using a scanning electron microscope (SEM) at 2,000x magnification. The examiner re-evaluated the mode of failure of all specimens a week afterwards to verify the reliability of the results.

### Data analysis

The data were analyzed using SPSS version 28 statistical software (Statistical Package for Statistical Science Inc., Chicago, IL, USA) at a 5% significance level. Descriptive statistics were performed to determine the mean and standard deviation (SD) values. The Kolmogorov-Smirnov test was used to determine the normality of the data distribution. Welch-analysis of variance (ANOVA) and the Games-Howell post-hoc comparison test were used to analyze the differences in the SBS, and Fisher’s Exacts test was used to evaluate the differences in the mode of failure between the tested groups.

## Results

The SBS of the surface treatment groups is presented in Table 2. No debonding occurred during specimen preparation. One-way ANOVA demonstrated significant difference in SBS between zirconia surface treatment methods (*p* < 0.001). Mechanical surface treatment by grinding produced the highest SBS, followed by air abrasion. Z-primer application typically resulted in a higher SBS regardless of the resin adhesive used (*p* < 0.001). When the primer was not applied, RelyX U200 resin adhesive resulted in a significantly higher SBS than Transbond XT (*p* < 0.001). In contrast, there was no significant difference in the SBS between the two resin adhesives when the Z-primer was applied (*p* = 0.998 for no mechanical surface treatment, *p* = 0.075 for sandblasting), except for the higher SBS of RelyX U200 when the specimens were subjected to grinding (*p* < 0.001). After grinding, using Z-primer and RelyX U200 resulted in a higher SBS compared with no primer application and using Transbond XT (*p* < 0.001).

**Table 2:** Shear bond strength values (MPa) after receiving different surface treatments and resin adhesives.

Surface treatment conditions		Types of resin adhesive	SBS values (mean ± SD)
Mechanical	Chemical		
No	No primer	Transbond XT	1.06 ± 0.13^h^
		RelyX U200	3.04 ± 0.50^f^
	Z-primer	Transbond XT	3.64 ± 0.42^ef^
		RelyX U200	3.68 ± 0.44^ef^
Air abrasion	No primer	Transbond XT	2.12 ± 0.26^g^
		RelyX U200	3.43 ± 0.43^ef^
	Z-primer	Transbond XT	3.93 ± 0.44^de^
		RelyX U200	4.18 ± 0.52^d^
Grinding	No primer	Transbond XT	4.07 ± 0.56^de^
		RelyX U200	4.63 ± 0.60^c^
	Z-primer	Transbond XT	6.89 ± 0.78^b^
		RelyX U200	8.36 ± 0.94^a^

Different lowercase letters indicate significant difference at *p* < 0.05.

**Fig. 3:**
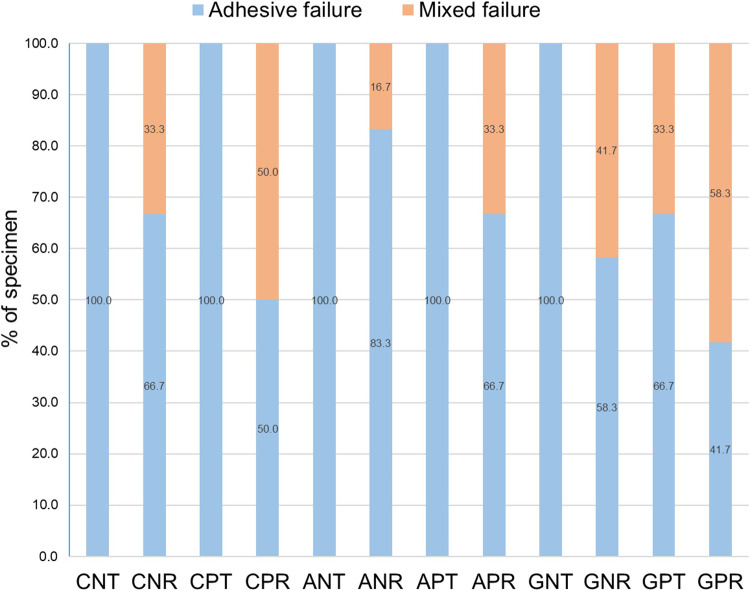
Mode of failure (%) of each surface treatment and resin adhesive (*n* = 12 per group). The first, second, and third capital letter indicated mechanical and chemical surface treatment, and types of resin adhesive, respectively. C no mechanical surface treatment, A air abrasion, G grinding, N no primer application, P primer application, T Transbond XT, R RelyX U200.

**Fig. 4:**
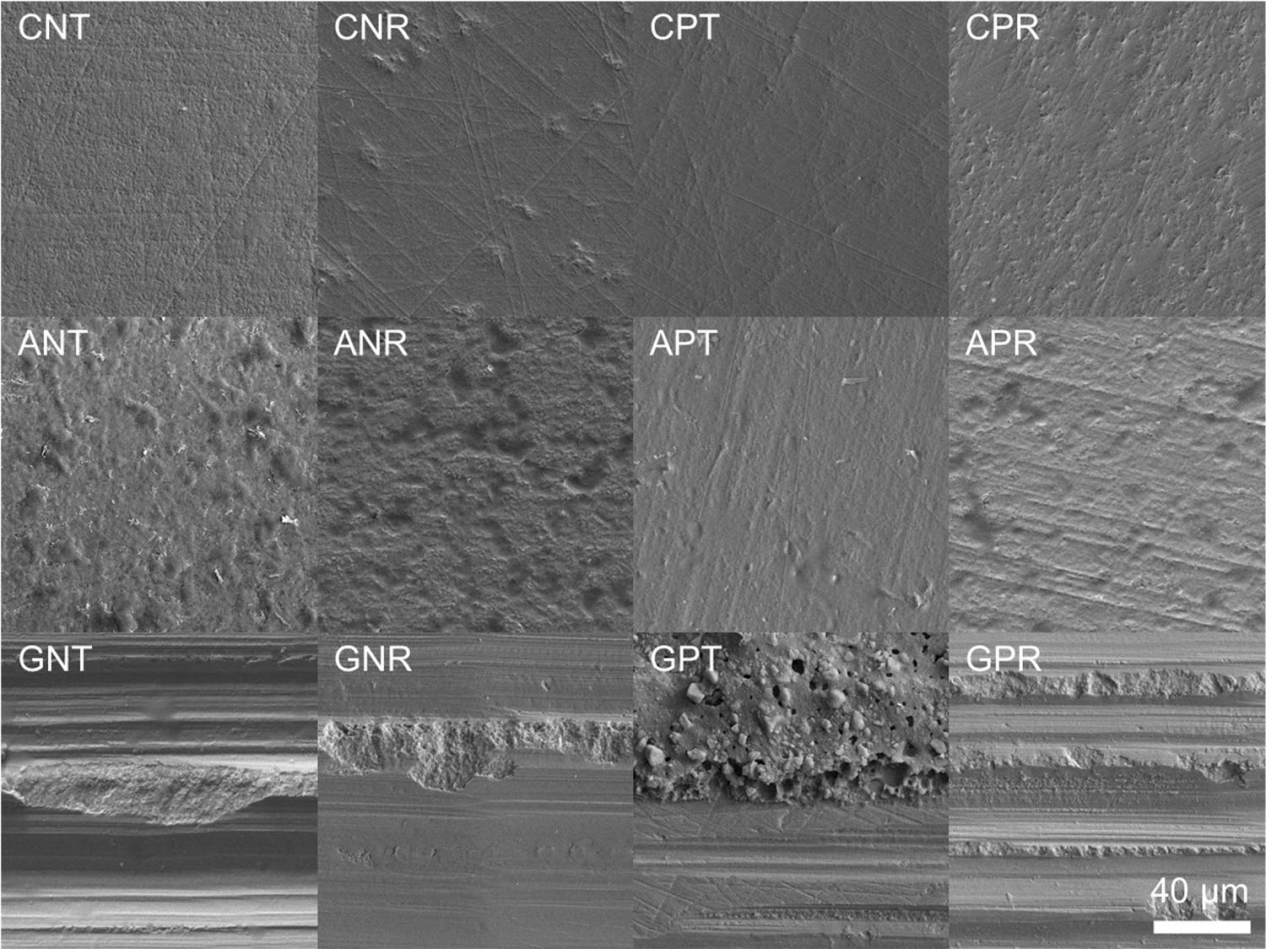
Scanning electron microscope (SEM) of the specimen after receiving different surface treatment. C no mechanical surface treatment, A air abrasion, G grinding, N no primer application, P primer application, T Transbond XT, R RelyX U200.

After loading, 100% adhesive failure at the zirconia–adhesive interface occurred when Transbond XT was applied without bur grinding, and when Transbond XT was used after grinding but without Z-primer application (Fig. 3). In contrast, the other groups demonstrated mixed failure at the YTZP–adhesive interface and cohesive failure within the resin adhesive. Table 3 showed the quantity of residual cement on the zirconia surface represented by ARI score. The group that combined grinding, primer application, and the use of RelyX U200 had the highest ARI score among all groups. However, failures at the ceramic-adhesive interface, indicated by ARI scores of 0 or 1, were prevalent in all groups. No ceramic fracture was observed. The SEM of the zirconia surface illustrated that air abrasion increased the surface roughness with a homogenous pattern, whereas grinding created a wavy-pattern of the dental bur and the remnant resin cement on the surface (Fig. 4).

**Table 3:** Distribution of specimens in each group (*n* = 12 per group) according to ARI score category.

Surface treatment condition	Number of specimens in each ARI category (*n*)				Sum ARI score
	0	1	2	3	
CNT	12	0	0	0	0
CNR	8	4	0	0	4
CPT	12	0	0	0	0
CPR	6	6	0	0	6
ANT	12	0	0	0	0
ANR	10	2	0	0	2
APT	12	0	0	0	0
APR	8	4	0	0	4
GNT	12	0	0	0	0
GNR	7	5	0	0	5
GPT	8	4	0	0	4
GPR	5	5	2	0	9

*ARI* Adhesive Remnant Index, *C* no mechanical surface treatment, *A* air abrasion, *G* grinding, *N* no primer application, *P* primer application, *T* Transbond XT, *R* RelyX U200.

## Discussion

The present study evaluated the combined effects of chemical and mechanical surface treatment, as well as resin adhesive types on the adhesion between metal brackets and zirconia restoration. Our results indicated that zirconia surface grinding, applying primer, and using RelyX U200 resin adhesive significantly improved the SBS between metal orthodontic bracket and zirconia. Therefore, the null hypothesis was rejected. The failure mode results also supported the quantitative findings, indicating that the groups with a relatively high bond strength demonstrated a combination of adhesive and cohesive failures within the resin adhesive.

Our study found that mechanical grinding provided the highest bond strength, followed by air abrasion, which is consistent with a previous study [11]. However, the improvement in SBS after air abrasion varied depending on primer application and resin adhesive type. This is likely because bur roughening exposes and create micropores on the zirconia substructure, which is different from other mechanical and chemical methods, such as air abrasion and hydrofluoric acid etching [11]. In vitro studies have shown that there is no significant difference in the SBS between metal brackets and zirconia crowns after air abrasion [10, 14], tribochemical silica coating, or carbon dioxide laser irradiation compared with no surface treatment [10]. Although a higher bond strength has been reported when using air abrasion compared with using a soflex disc [16], this might be due to the smaller grit size in the solflex disc, which is insufficient to roughen the zirconia surface.

The chemical surface treatment using zirconia primer application demonstrated a positive effect on improving the adhesion of metal bracket to zirconia, as seen in previous in vitro studies [17, 18]. This is due to the combined mechanical and chemical bonding mechanisms of the primer. The primer increases the wettability of the resin adhesive and reduces the contact angle between the zirconia and resin adhesive, resulting in greater flow and adaptation between the two layers [24]. Furthermore, the active phosphate monomer in the 10-methacryloxydecyl dihydrogen phosphate (MDP) component of the Z-primer can chemically bond with the oxide layer of zirconia [25, 26]. Therefore, primer application generally improves the adhesion of metal brackets to zirconia, regardless of the mechanical surface treatment method and type of resin adhesive used.

Without primer application, RelyX U200 resin adhesive achieved better adhesion between the metal brackets and Zirconia compared with Transbond XT. Transbond XT has been specifically recommended as a two-step adhesive for bonding metal or ceramic bracket with tooth structure [27], whereas RelyX U200 is a universal resin cement for bonding between various restorative materials and tooth structure [28]. Two possible mechanisms that lead to the greater effectiveness of RelyX U200 can be explained by mechanical and chemical bonding. First, RelyX U200 has a lower viscosity and greater flowability than Transbond XT [29, 30], resulting in greater adaptation with the zirconia surface and a higher bond strength [31]. Second, the MDP component in RelyX U200 contains a phosphate functional group that can form a chemical bond with the hydroxyl group in zirconia restorations. In contrast, Transbond XT’s main component, bisphenol-A-diglycidyl methacrylate (BisGMA), cannot chemically bond with zirconia [15]. A previous in vitro study also showed that using a resin adhesive containing MDP resulted in a higher bond strength with zirconia than using the BisGMA-containing adhesive [32]. Another study found no difference in SBS between Panavia and RelyX U200, likely because both resin adhesives contain MDP [16].

Based on our findings, a synergistic effect was observed when combining mechanical and chemical surface treatment with a specific type of resin adhesive. When performing zirconia surface grinding in conjunction with primer application and using RelyX U200, improved adhesion between the metal brackets and zirconia was observed, as supported by the highest bond strength and ARI score. This can be attributed to the increased flow and adaptation between the two layers due to the micromechanical interlocking created through the ground surface. In addition, the MDP in the primer and resin adhesive may create chemical bonds with zirconia restorations. Grinding zirconia can result in increased surface roughness, potentially causing color instability due to color infiltration [33]. This is a notable concern, especially when working with zirconia restorations for anterior teeth. Therefore, it is important to emphasize the need for proper polishing techniques, including the use of appropriate polishing burs and optimal polishing durations [34], to maintain color stability and prevent material wear and deterioration [33, 34].

The mode of failure results revealed that the cohesive failure within the resin adhesive was the most common failure type when using the primer and RelyX U200, particularly in combination with grinding. Therefore, the adhesion between metal brackets and zirconia can be improved through a combination of mechanical interlocking and chemical bonding created by surface roughening and using an MDP-containing primer and adhesive. Despite a high SBS between the metal bracket and zirconia, the force required for bracket removal is unlikely to result in zirconia breakage. This is because debonding primarily occurs within the resin cement or at the interface between the metal bracket and resin cement.

Our findings suggest that selecting the appropriate surface treatment protocol and resin adhesive materials can improve the adhesion of orthodontic brackets to zirconia restorations. However, some limitations are noted in this study. The findings from this in vitro study may provide different results in clinical performance because the oral environment may affect the adhesion. Further clinical studies using a long-term follow-up are suggested to verify the clinical significance of the present findings. Moreover, it is imperative to explore the impact of optimizing bur types for grinding, grinding speed, and the use of water coolant on the bond strength between metal brackets and zirconia restorations.

## Conclusion

Our findings indicated that mechanical surface treatment using bur grinding, combined with applying an MDP-containing primer and resin adhesive, enhances the SBS between zirconia and metal orthodontic brackets. These combinations also result in a more cohesive failure within resin adhesive, rather than adhesive failure between the two layers.

### Supplementary information

Table 1, Table 2, Table 3

## Data Availability

Data is available upon request to the corresponding author.
