# Supplementary material for: Mechanical and chemical surface treatment enhances bond strength between zirconia and orthodontic brackets: an in vitro study
Source: BDJ Open. 2023 Dec 8;9:53. doi: 10.1038/s41405-023-00180-6 (PMC10709639; doi:10.1038/s41405-023-00180-6)
Supplement: Supplementary file 1 — Table 1, Table 2, Table 3 [file 41405_2023_180_MOESM1_ESM.docx]

**TABLES**

**Table 1.** Materials used in this study

| **Material type** | **Brand** | **Manufacturer** | **Main composition** |
| --- | --- | --- | --- |
| Mechanical surface treatment | | | |
| Aluminum oxide particle (Al_2_O_3_) |  |  | 50-µm aluminum oxide particle |
| Diamond bur | Intensive | Intensive SA, Grancia, Switzerland | 106-µm diameter diamond grit size |
| Chemical surface treatment | | | |
| Primer | Z-Prime^TM^ Plus | Bisco, Inc. USA | - Bis-GMA  - MDP  - 2-hydroxyethyl methacrylate, ethanol |
| Resin adhesives | | | |
| Dual cure self-adhesive | RelyX^TM^ U200 | 3M EPSE, USA | Base:  - methacrylate monomers, and those containing phosphoric acid groups  - silanated fillers, Initiators, stabilizers, rheological additives.  Catalyst:  - Methacrylate monomers  - silanated fillers, Alkaline stabilizers, rheological additives. |
| Light cure self-adhesive | Transbond^TM^ XT | 3M EPSE, USA | Adhesive:  - bis-GMA, silane,  - bisphenol A bis (2-hydroxyethyl ether) dimethacrylate  - diphenyliodonium hexafluorophosphate  Primer:  - bis-GMA, TEGDMA  - triphenyl antimony, 4-(dimethylamino)-benzeneethanol  - DL-Camphorquinone, Hydroquinone |

Bis-GMA, bisphenol-A-diglycidylmethacrylate; MDP: 10-methacryloxydecyl dihydrogen phosphate; TEGDMA, triethyleneglycol dimethacrylate

**Table 2.** Shear bond strength values (MPa) after receiving different surface treatments and resin adhesives.

| **Surface treatment conditions** | | **Types of resin adhesive** | **SBS values**  **(mean ±SD)** |
| --- | --- | --- | --- |
| **Mechanical** | **Chemical** |  |  |
| No | No primer | Transbond XT | 1.06 ±0.13^h^ |
|  |  | RelyX U200 | 3.04 ±0.50^f^ |
|  | Z-primer | Transbond XT | 3.64 ±0.42^ef^ |
|  |  | RelyX U200 | 3.68 ±0.44^ef^ |
| Air abrasion | No primer | Transbond XT | 2.12 ±0.26^g^ |
|  |  | RelyX U200 | 3.43 ±0.43^ef^ |
|  | Z-primer | Transbond XT | 3.93 ±0.44^de^ |
|  |  | RelyX U200 | 4.18 ±0.52^d^ |
| Grinding | No primer | Transbond XT | 4.07 ±0.56^de^ |
|  |  | RelyX U200 | 4.63 ±0.60^c^ |
|  | Z-primer | Transbond XT | 6.89 ±0.78^b^ |
|  |  | RelyX U200 | 8.36 ±0.94^a^ |

Different lowercase letters indicate significant difference at p < 0.05.

**Table 3.** Distribution of specimens in each group (n=12 per group) according to ARI score category.

| **Surface treatment condition** | **Number of specimens in each ARI category (n)** | | | | **Sum ARI score** |
| --- | --- | --- | --- | --- | --- |
|  | **0** | **1** | **2** | **3** |  |
| CNT | 12 | 0 | 0 | 0 | 0 |
| CNR | 8 | 4 | 0 | 0 | 4 |
| CPT | 12 | 0 | 0 | 0 | 0 |
| CPR | 6 | 6 | 0 | 0 | 6 |
| ANT | 12 | 0 | 0 | 0 | 0 |
| ANR | 10 | 2 | 0 | 0 | 2 |
| APT | 12 | 0 | 0 | 0 | 0 |
| APR | 8 | 4 | 0 | 0 | 4 |
| GNT | 12 | 0 | 0 | 0 | 0 |
| GNR | 7 | 5 | 0 | 0 | 5 |
| GPT | 8 | 4 | 0 | 0 | 4 |
| GPR | 5 | 5 | 2 | 0 | 9 |

ARI, Adhesive Remnant Index; C, no mechanical surface treatment; A, air abrasion; G, grinding;

N, no primer application; P, primer application; T, Transbond XT; R, RelyX U200
